# Warm up with music and visual feedback can effect Wingate performance in futsal players

**DOI:** 10.1186/s13102-022-00601-3

**Published:** 2022-12-07

**Authors:** Serdar Bayrakdaroğlu, Özgür Eken, Fatma Hilal Yagin, Ramazan Bayer, Mehmet Gulu, Zeki Akyildiz, Hadi Nobari

**Affiliations:** 1 Movement and Training Science, Gumushane University, Gumushane, Turkey; 2 Department of Physical Education and Sport Teaching, Inonu University, Malatya, Turkey; 3 Department of Biostatistics and Medical Informatics, Faculty of Medicine, Inonu University, Malatya, Turkey; 4 Faculty of Health Sciences, Malatya Turgut Ozal University, Malatya, Turkey; 5 Department of Coaching Education, Faculty of Sport Sciences, Kirikkale University, 71450 Kirikkale, Turkey; 6 Sports Science Department, Gazi University, Ankara, Turkey; 7 Department of Exercise Physiology, Faculty of Educational Sciences and Psychology, University of Mohaghegh Ardabili, Ardabil, 56199-11367 Iran; 8 Faculty of Sport Sciences, University of Extremadura, 10003 Cáceres, Spain; 9 Department of Motor Performance, Faculty of Physical Education and Mountain Sports, Transilvania University of Braşov, 500068 Braşov, Romania

**Keywords:** Warm up, Music, Visual feedback, Wingate

## Abstract

**Purpose:**

Visual feedback and music while warm up may help elicit peak performance, but its effect during the Wingate anaerobic test (WAnT) in futsal players is unexplored. Therefore, the aim of this study was to examine the effects of music and visual stimulus on WAnT performance in futsal players.

**Methods:**

The study included 17 young male futsal players (age, 21.53 ± 1.32 years; height, 177.59 ± 5.75 cm; 73.71 ± 9.31 kg; BMI 23.342 ± .47). The WAnT was administered using three distinct protocols: with music (M), without music (NM), and with music and visual feedback (MV) following a 10-min warm-up at 72-h intervals. After WAnT, the following parameters were evaluated: (a) peak power output: PPO (W), (b) relative peak power output: RPPO (W/kg), (c) mean power output: MPO (W), (d) relative mean power output: RMPO (W/kg), (e) fatigue index: FI (%), and (f) minimum power output MIPO (W).

**Results:**

MV indicated that futsal players' WAnT values, including PPO, RPPO, MPO, RMPO, and MIPO improved more than with other protocols (*p* < 0.05). However, there was no significant difference between protocols for FI (%) WAnT values (*p* > 0.05).

**Conclusions:**

Listening to music and watching visual feedback while warm up before performing WAnT performance suggest to coaches and futsal players.

## Background

Futsal requires anaerobic power and capacity such as sprinting, sudden change of direction and jumping in the field. Many studies have shown that listening to music while warming up significantly improves anaerobic exercise performance [1–4]. Various training methods and ergogenic supplements are being researched to increase anaerobic performance. In a study, Chtorou et al. [2] found that power output increased in male sprinters during the 30-s Wingate Anaerobic Test (WAnT) after a 10-min warm-up with and without music. It has been reported in many exercises in the literature that methods such as warm up, stretching, music and visual feedback before competitions and training are necessary to improve athletic performance [2, 5–10]. Pre-exercise warm-up, a common technique, is defined as necessary to maximize the athlete's performance in a variety of sports and physical activities by changing the body's physiological mechanisms (muscle temperature, nerve conduction velocity, increased blood flow). In addition, the increased anaerobic metabolism induced by passive and active warm up may have metabolic, neurological, and psychological consequences, such as an increase in oxygen uptake kinetics and post-activation potentiation [11]. In addition, warm up before exercise or competition can enhance the effectiveness of muscle glycolysis and the breakdown of high-energy phosphates during exercise by increasing muscle temperature, muscular metabolism, and muscle fiber conduction velocity. This causes a positive increase in the activity of VO_2_ kinetics following the prior contraction of a muscle. Modulating the cross-bridge cycle rate and oxygen uptake kinetics can therefore increase muscle function [12].

Numerous sportsmen like listening to music during warm up and work out at a high level of intensity. However, research on the effects of music on athletic performance has yielded mixed results, with some suggesting that the timing and type of music may influence the anaerobic performance response [13]. Music is an external resource that can be used to enhance the ergogenic effect of a wide variety of exercise modes and intensities [14, 15]. Additionally, it has demonstrated that changes in the mood, motivation, warm-up speed, and arousal of music can result in performance gains [14, 16]. Preference for music has been shown to be a significant factor in determining music's ergogenic potential [14, 15]. However, how preference affects the aforementioned mechanisms, particularly during anaerobic exercise, is unknown. The relationship between music and anaerobic exercise has been studied primarily through the use of predetermined music, with mixed results [17–19]. Besides visual feedback, which is equally critical for athletic performance, can aid in determining peak performance throughout many types of strength and power testing, but its effect on anaerobic Wingate is worth investigating [20].

Various tests are used to evaluate anaerobic performance. The 30-s Wingate anaerobic test (WAnT) is one of the most commonly used tests to assess lower body anaerobic performance. WAnT is considered the gold standard test to evaluate anaerobic performance in many sports disciplines [21–24]. Other anaerobic tests can measure peak power; these tests are vertical jump tests, standing long jump test, and Bosco repeated jump tests [25–27].

Many studies have shown that listening to music while warm up significantly improves anaerobic exercise performance [1–4]. Studies have reported that listening to music provides an increase in peak and average power values in the 30 s WAnT performance [2, 4]. Brooks et al. [28] reported increases in peak and average power during the 2 × 30 s WAnT in performance with music compared to performance without music. Simpson et al. [18] reported that using music during a 400 m sprint has a positive effect on performance. A systematic review and meta-analysis showed that listening to music during WAnT may increase anaerobic exercise performance physiologically, although the reasons remain speculative [13]. However, other researchers' findings showed little or no improvement in anaerobic performance with warm-up music [3, 4, 17]. The reasons for the differences between the findings are not entirely clear. For this reason, it needs to be investigated.

Additionally, visual input has been shown to influence athletic performance [20, 29]. While visual feedback has been shown to be beneficial in determining the short-term maximum effort required to obtain the highest isokinetic force production, its effect on maximum force or power output in other types of tests has not been well explained [30]. Additionally, studies have demonstrated that the provision of visual feedback results in increased performance during short-term maximum test runs, which are typical of strength and power testing [31, 32].

With the results obtained from the WAnT, sports scientists and practitioners form the roadmap of athletes. It is very important that the WAnT, which provides very detailed information to determine the training programs of the athletes and the deficiencies in the athletes, is measured at the maximum level. In order to maximize the results of the tests, it is very important to try to increase the test performance output with feedback and music during the test. While additional research is needed to determine the impacts of music and visual feedback while warm up on WAnT performance, the preference of futsal players and a dearth of literature to support such an effect are deemed significant. The research that results in such a design can aid coaches in determining the most appropriate scenario and in utilizing visual feedback and music to improve futsal players' match readiness. Additionally, music and visual feedback can be used to improve the performance of WAnT values such as PPO, RPPO, MPO, RMPO, and FI. However, it is unknown whether warming music and visual stimuli affect WAnT performance and whether possible effects occur in psychological or psychophysiological mechanisms. Visual feedback and music while warm up may help elicit peak performance, but its effect during the WAnT in futsal players is unexplored. Therefore, the aim of this study was to examine the effects of music and visual stimulus on WAnT performance in futsal players. Our hypothesis was that music and visual stimuli will improve anaerobic performance.

## Method and material

### Participants

The study included 17 male futsal players aged 18–25 who had been active for at least four years and exercised regularly 5 days a week (age, 21.53 ± 1.32 years; height, 177.59 ± 5.75 cm; 73.71 ± 9.31 kg; BMI 23.34 ± 2.47). The study group was determined using the power analysis program G*Power (version 3.1.9.3, Germany). As a result of the power analysis (confidence interval = 0.95, alpha = 0.05, power (1-beta) = 0.80, and effect size = 0.33), it was determined that at least 17 futsal players should be included in the study [33]. The criteria for including volunteer futsal players in the research were as follows: (a) they must have participated in licensed sports for at least four years; (b) they must not have a history of disability that would impair the study's outcome; (c) they must commit to regular participation in the study; and (d) they must obey the investigators' commands throughout the study. The exclusion criteria of the study were reported to the volunteers as having problems such as disability that would affect the result of the study and having used stimulant drugs until the day before the study. All futsal players were informed of the study's requirements and risks, and signed an informed consent form indicating their voluntary participation in the study. The futsal players were instructed to maintain their normal physical activity throughout the study, but to abstain from strenuous activities for 24 h prior to the study. Throughout the study period, futsal players were instructed not to use any drugs (anabolic steroids, other hormones, metabolic modulators, diuretics, or non-steroidal anti-inflammatory drugs NSAIDs, for example) or receive any medical treatment (blood transfusion, blood donation). Additionally, the futsal players were asked to monitor their sleep habits prior to the study. Prior to initiating the study, the Malatya Inonu University Clinical Research Ethics Committee approved it (Ethics Committee Protocol Number 2021/2719).

### Experimental design

**Fig. 1:**
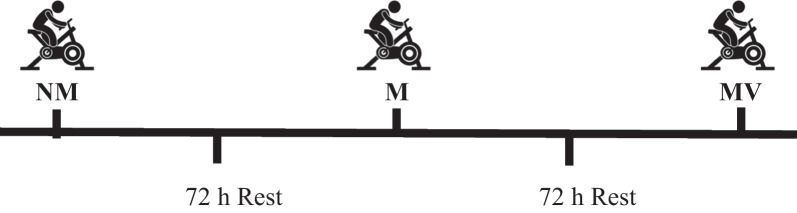
Wingate anaerobic strength test (WAnT) protocol design

Futsal players were recruited from a single group. Three distinct protocols were used to conduct the measurements, with each protocol being applied 72 h apart [34]. The familiarization phase began one week prior to the study by providing information about the futsal players during the test process. To avoid the circadian rhythm's effect on each protocol, all tests were conducted at the same time of day (05:00–07:00 pm) [35]. As a result, the following protocols were established (Fig. 1).

- No music and visual feedback phase (NM); First, the warm-up was implemented by pedaling in WAnT for 10 min at a heart rate corresponding to 40% of the determined HRR. the intensity of the warm- up was determined by calculating 40% of the HRR using the formula of Karvonen. The calculation of the Karvonen formula was as follows: Target heart rate = exercise intensity × (maximum heart rate − resting heart rate) + resting heart rate [36, 37]. The heart rates of the subjects were monitored during the 10-min warm-up using a Polar RS400 watch. After 10 min of warm-up WAnT was applied to subjects.
- Music phase (M); the same procedure above was used to determine the intensity of warm up. In music condition, subjects applied to 10 min of warm up by listening to music (120–140 bpm) rather than just warm up by pedaling [38, 39]. After 10 min of warm-up, the WanT was applied while maintaining the same musical conditions.
- Music and visual feedback phase (MV); the same procedure was used to determine the intensity of warm up. In music and visual feedback condition, subjects applied to 10 min of warm up by listening to music (120–140 bpm) and watching a video that displaying athletes who perform high intensity exercises (indoor and outdoor) such as sprinting on a hill or lifting heavy weights to motivate subjects. After 10 min of warm-up, the WAnT was applied while maintaining the same conditions.

### Wingate anaerobic strength test (WAnT) protocol

The Wingate Anaerobic Power Test (WAnT) was used to determine the participants' anaerobic capacity and power. After thoroughly briefing the subjects on the test procedure, the test was conducted using a computer ergometer (Monark 839E Sweden) equipped with compatible software and connected to a specially modified computer for the leg. The WAnT is the most widely used bicycle ergometer test for determining anaerobic performance in which the subject pedals as quickly as possible against a resistance determined by body mass [40, 41]. For each test, participants were seated on the bicycle ergometer and seat height, pedal position, and upper body position were adjusted to the participant's satisfaction. To prevent futsal players from slipping off the pedals during the test, finger clips were used. These adjustments were made for each athlete and maintained throughout all protocols. Throughout the test, futsal players were given strong verbal motivational stimuli to perform at their peak. The load was determined for each athlete in the WAnT test according to their body mass (0.087 kg − 1 body mass) using BarOr [42] optimization tables, and the athlete attempted to pedal at a high speed for 30 s against the resistance created by the load. The pedal numbers were recorded every 5 s, and the five major parameters of WAnT were evaluated in the study by computing the absolute and relative values of all power parameters using computer software: (a) peak power output: PPO (W), (b) relative peak power output: RPPO (W/kg), (c) mean power output: MPO (W), (d) relative mean power output: RMPO (W) /kg), and (e) fatigue index: FI (%). Additionally, the following formula was used to determine the FI (%) values [40, 42].

Wingate Anaerobic Strength Test (WAnT) Protocol

$${\text{Fatigue}}\;{\text{index}}\left( \% \right) = \,\left( {{\text{peak}}\;{\text{power}} - {\text{lowest}}\;{\text{power}}} \right)/{\text{peak}}\;{\text{power}}) \, \times \, 100$$

### Statistical analysis

In this investigation, the assumption of normal distribution for quantitative data was checked with the Shapiro Wilk test. Since the quantitative data showed a normal distribution, they were expressed as the mean and standard deviation. The effect of different protocols (NM, M, and MV) on measurement times was determined using the Repeated Measures ANOVA test. Mauchly's sphericity test was used to test the homogeneity of variances and Greenhouse–Geisser correction was applied when necessary. Partial eta-squares $\left( {\eta_{p}^{2} } \right)$ were calculated to examine the magnitude of the effect between groups. When statistically significant differences were discovered between study protocols, multiple comparison analyzes were performed using the Bonferroni method. *p* < 0.05 was considered significant. All statistical analyzes were performed using IBM SPSS Statistics for Windows version 28.0 (New York; USA) software and graphs using GraphPad Prism 9.4.1 software.

## Results

**Fig. 2:**
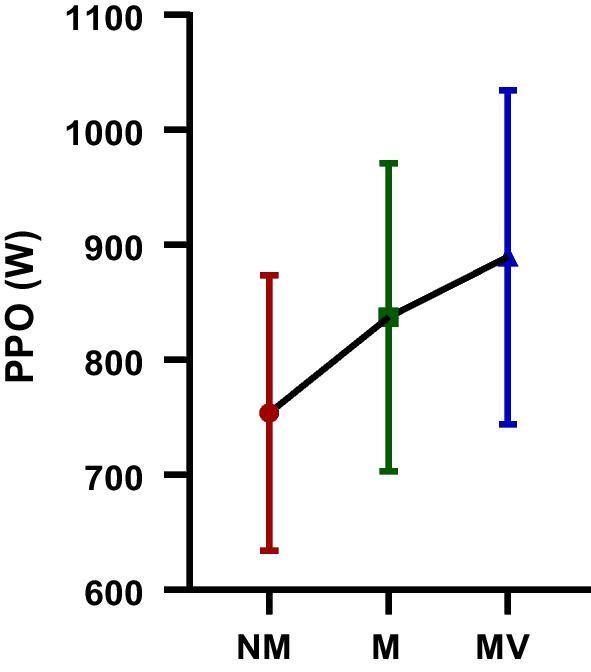
The peak power output (PPO (W)): The Wingate anaerobic power test (WAnT) values after no music and visual feedback (NM), music (M) and music and visual feedback (MV) protocols participating in the study; *: statistical significance

The difference between the PPO (W) WAnT values of the futsal players participating in the study following the NM, M, and MV protocols is depicted in Table 1 and Fig. 2. The PPO (W) WAnT values observed following the MV (889.73 ± 145.22) protocol are greater than those observed following the M (837.33 ± 134.16) and NM (754.07 ± 119.93) protocols, respectively. Additionally, NM, M, and MV were found to be among the PPO (W) WAnT values following all protocols indicating a statistically significant difference [F = 47.53; *p* < 0.001; ${\eta }_{p}^{2}$=0.75].

**Table 1:** PPO (W) WAnT values after NM, M and MV protocols participating in the study

Groups	Mean ± SD	Between measurements
		F value
		*p* value
		$${\mathbf{\eta_{p}^{2}}}$$
PPO (W) NM	754.07 ± 119.93	F = 47.53 *p* < 0.001 $\eta_{p}^{2}$ = 0.75
PPO (W) M	837.33 ± 134.16	
PPO (W) MV	889.73 ± 145.22	

*PPO*, peak power output; *NM*, no music and visual feedback phase; *M*, music phase; *MV*, music and visual feedback phase; *SD*, standard deviation; $\eta_{p}^{2}$: Partial eta-squares.

**Fig. 3:**
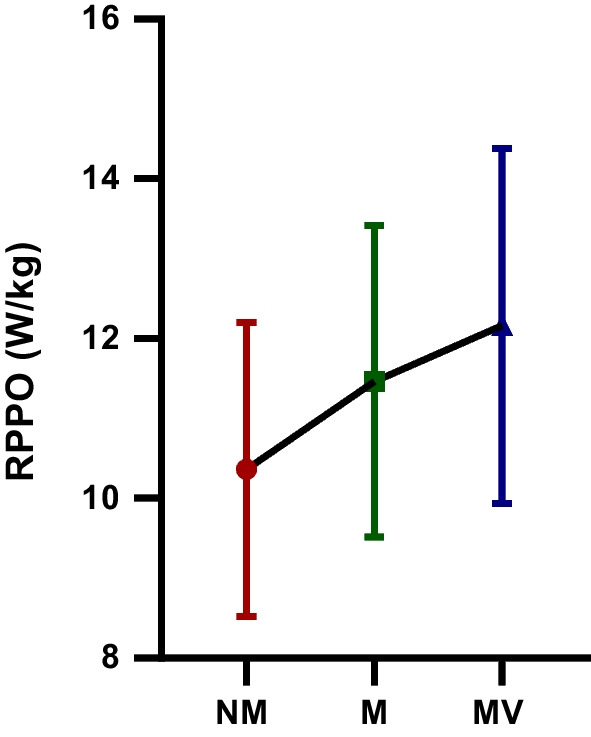
The relative peak power output (RPPO (W/kg)): The Wingate anaerobic power test (WAnT) values after no music and visual feedback (NM), music (M) and music and visual feedback (MV) protocols participating in the study; *: statistical significance

The difference in RPPO (W/kg) WAnT values between the NM, M, and MV protocols is shown in Table 2 and Fig. 3. There was a statistically significant difference in RPPO (W/kg) WAnT values between the NM, M, and MV protocols [F = 38.95; *p* < 0.001; $\eta_{p}^{2}$ = 0.71]. The RPPO (W/kg) WAnT values observed after the MV (12.16 ± 2.22) protocol were significantly higher than the M (11.46 ± 1.95) and NM (10.36 ± 1.84) protocols (*p* < 0.05).

**Table 2:** RPPO (W/kg) WAnT values after NM, M and MV protocols participating in the study

Groups	Mean ± SD	Between measurements
		F value
		*p* value
		$${\mathbf{\eta_{p}^{2}}}$$
RPPO (W/kg) NM	10.36 ± 1.84	F = 38.95 *p* < 0.001 $\eta_{p}^{2}$ = 0.71
RPPO (W/kg) M	11.46 ± 1.95	
RPPO (W/kg) MV	12.16 ± 2.22	

*RPPO*, relative peak power output; *NM*, no music and visual feedback phase; *M*, music phase; *MV*, music and visual feedback phase; *SD*, standard deviation; $\eta_{p}^{2}$: Partial eta-squares.

**Fig. 4:**
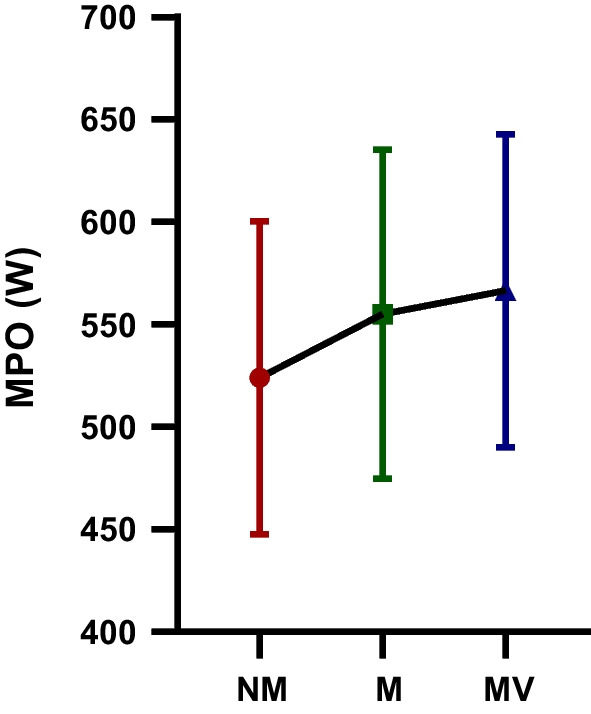
The mean power output (MPO (W)): The Wingate anaerobic power test (WAnT) values after no music and visual feedback (NM), music (M) and music and visual feedback (MV) protocols participating in the study; *: statistical significance

The difference between the MPO (W) WAnT values of the futsal players participating in the study following the NM, M, and MV protocols is depicted in Table 3 and Fig. 4. According to the results of the study, there was a statistically significant difference in MPO (W) WAnT values between the NM, M, and MV protocols [F = 20.12; *p* < 0.001; $\eta_{p}^{2}$ = 0.56].The MPO (W) WAnT values observed after the MV (566.57 ± 76.38) protocol were greater than the MPO (W) WAnT values observed after the M (555.03 ± 80.25) and NM (524.08 ± 76.21) protocols, respectively. In addition, Post-hoc analyzes showed a statistically significant difference in terms of MPO (W) WAnT between the NM - M (*p* = 0.002) and NM - MV (*p* = 0.001) protocols.

**Table 3:** MPO (W) WAnT values after NM, M and MV protocols participating in the study

Groups	Mean ± SD	Between measurements
		F Value
		*p* Value
		$${\mathbf{\eta_{p}^{2}}}$$
MPO (W) NM	524.08 ± 76.21	F = 20.12 *p* < 0.001 $\eta_{p}^{2}$ = 0.56
MPO (W) M	555.03 ± 80.25	
MPO (W) MV	566.57 ± 76.38	

*MPO*, mean power output; *NM*, no music and visual feedback phase; *M*, music phase; *MV*, music and visual feedback phase; *SD*, standard deviation; $\eta_{p}^{2}$: Partial eta-squares.

**Fig. 5:**
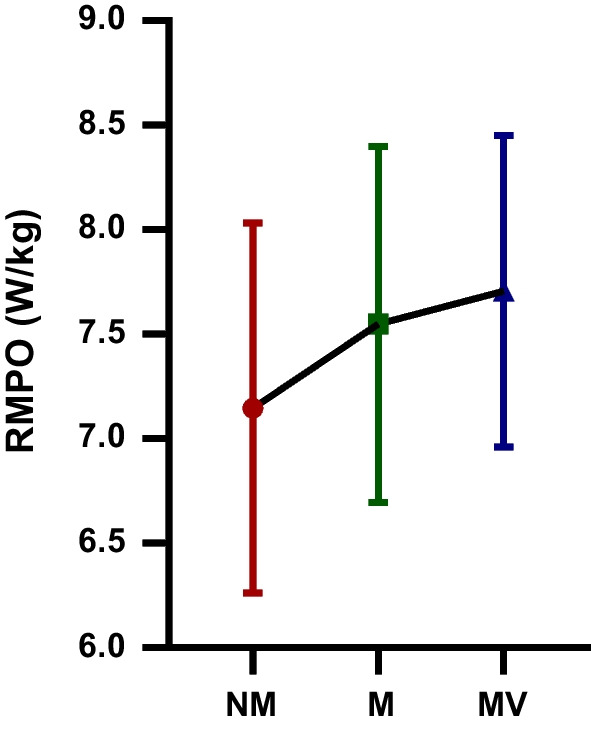
The relative mean power output (RMPO (W/kg)): The Wingate anaerobic power test (WAnT) values after no music and visual feedback (NM), music (M) and music and visual feedback (MV) protocols participating in the study; *: statistical significance

The difference in the RMPO (W/kg) WAnT values of the futsal players participating in the study following the NM, M, and MV protocols is depicted in Table 4 and Fig. 5. When comparing the RMPO (W/kg) WAnT values after the NM, M, and MV protocols [F = 18.36; *p* < 0.001; $\eta_{p}^{2}$ = 0.53], there was a statistically significant difference between the NM - M (*p* = 0.004) and NM - MV (*p* = 0.001) protocols. The RMPO (W/kg) WAnT values observed following the MV (7.71 ± 0.74) protocol were found to be superior to the RMPO (W/kg) WAnT values observed following the M (7.55 ± 0.85) and NM (7.15 ± 0.88) protocols, respectively.

**Table 4:** RMPO (W) WAnT values after NM, M and MV protocols participating in the study

Groups	Mean ± SD	Between measurements
		F value
		*p* value
		$${\mathbf{\eta_{p}^{2}}}$$
RMPO (W) NM	7.15 ± 0.88	F = 18.36 *p* < 0.001 $\eta_{p}^{2}$ = 0.53
RMPO (W) M	7.55 ± 0.85	
RMPO (W) MV	7.71 ± 0.74	

*RMPO*, relative mean power output; *NM*, no music and visual feedback phase; *M*, music phase; *MV*, music and visual feedback phase; *SD*, standard deviation; $\eta_{p}^{2}$: Partial eta-squares.

**Fig. 6:**
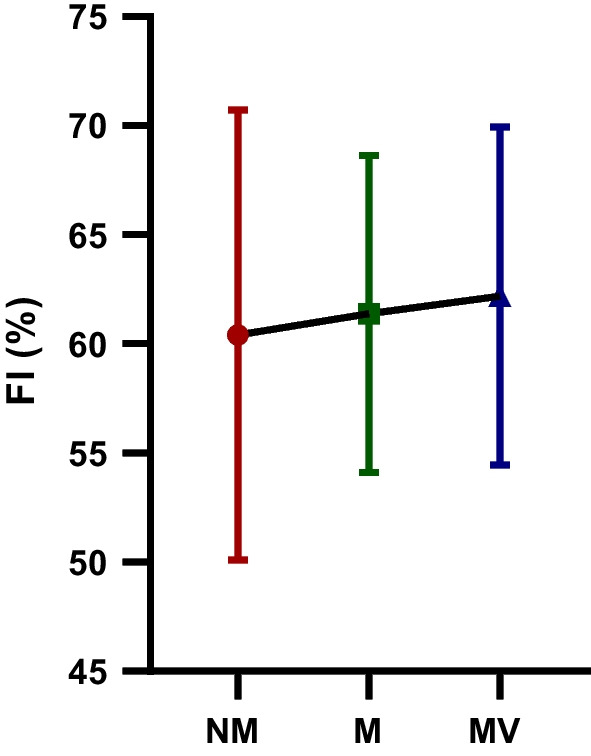
The fatigue index (FI (%)): The Wingate anaerobic power test (WAnT) values after no music and visual feedback (NM), music (M) and music and visual feedback (MV) protocols participating in the study; ns: no – significance

The difference in the FI (%) WAnT values of the futsal players participating in the study following the NM, M, and MV protocols is depicted in Table 5 and Fig. 6. The FI (%) WAnT values observed following the MV (62.20 ± 7.74) protocol were compared to the FI (%) WAnT values observed following the M (61.38 ± 7.26) and NM (60.40 ± 10.30) protocols, respectively. There was no statistically significant difference in FI (%) WAnT values following the NM, M and MV protocols [F = 0.41; *p* = 0.66; $\eta_{p}^{2}$ = 0.02]. So the protocols did not affect the FI (%) WAnT values.

**Table 5:** FI (%) WAnT values after NM, M and MV protocols participating in the study

Groups	Mean ± SD	Between measurements
		F value
		*p* value
		$${\mathbf{\eta_{p}^{2}}}$$
FI (%) NM	60.40 ± 10.30	F = 0.41 *p* = 0.66 $\eta_{p}^{2}$ = 0.02
FI (%) M	61.38 ± 7.26	
FI (%) MV	62.20 ± 7.74	

*FI*, fatigue index; *NM*, no music and visual feedback phase; *M*, music phase; *MV*, music and visual feedback phase; *SD*, standard deviation; $\eta_{p}^{2}$: Partial eta-squares.

**Fig. 7:**
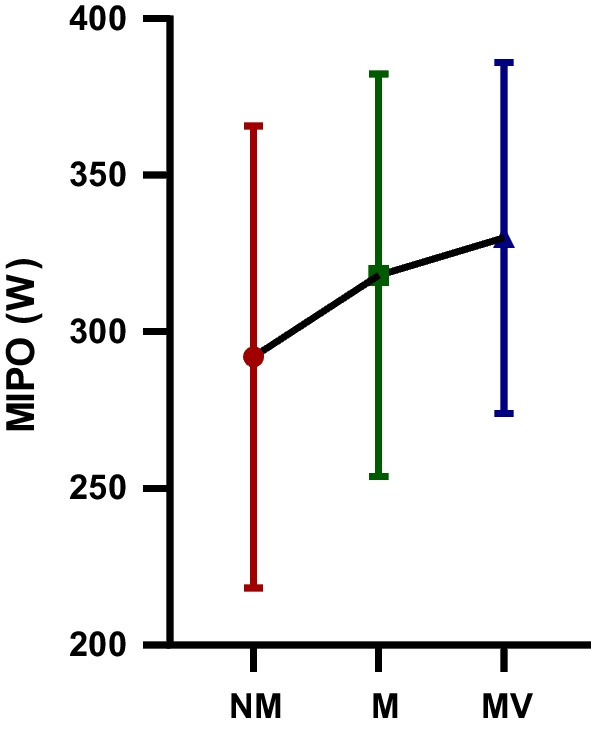
The minimum power output (MIPO (W)): The Wingate anaerobic power test (WAnT) values after no music and visual feedback (NM), music (M) and music and visual feedback (MV) protocols participating in the study; *: statistical significance; ns: no – significance

The difference in FI (%) WAnT values of the futsal players participating in the study following the NM, M, and MV protocols are shown in Table 6 and Fig. 7. A statistically significant difference was observed in MIPO (W) WAnT values following the NM, M and MV protocols [F = 4.55; *p* = 0.01; $\eta_{p}^{2}$ = 0.22]. MIPO (W) WAnT values observed after MV protocol (330.01 ± 56.09) were significantly higher compared to MIPO (W) WAnT values observed following NM (292.02 ± 73.72) protocols (*p* = 0.01). However, there was no statistically significant difference between the M-MV and M-MV protocols in terms of MIPO (W) WAnT values.

**Table 6:** MIPO (W) WAnT values after NM, M and MV protocols participating in the study

Groups	Mean ± SD	Between measurements
		F value
		*p* value
		$${\mathbf{\eta_{p}^{2}}}$$
MIPO (W) NM	292.02 ± 73.72	F = 4.55 *p* = 0.01 $\eta_{p}^{2}$ = 0.22
MIPO (W) M	318.13 ± 64.21	
MIPO (W) MV	330.01 ± 56.09	

*MIPO*, minimum power output; *NM*, no music and visual feedback phase; *M*, music phase; *MV*, music and visual feedback phase; *SD*, standard deviation; $\eta_{p}^{2}$: Partial eta-squares

## Discussion

The purpose of this study was to investigate warm up with music and visual feedback can effect WAnT results in futsal players. The major conclusion of this study was that significant linear increases in Wingate's PPO, RPPO, MPO, RMPO, and FI findings were obtained, particularly after MV.

Examining the literature, it is believed that the activation performed prior to Wingate is considered a warm-up phase and motivational effect that influences performance [2, 4, 13, 14, 17, 28, 43–45]. Brooks et al. [28] in their research, reported increases in mean and peak power during 2 × 30 s WAnT when comparing music to no-music conditions. In another study, Simpson et al. [18] reported that they performed better using music without using music during a 400 m sprint. These results support our findings. Contrary to our findings, Pujol et al. [19] found no changes in power output during 3 × 30 s WAnT when comparing music to no music. Atan et al. [17] similarly, the research of 's studies could not find the effect of music on performance. Studies have been carried out on its effect on performance using music determined in previous studies. Music preference can also be a factor in this situation. As a matter of fact, it has been found in studies that listening to preferred music is more stimulating than non-preferred music, improves perceived exertion (RPE) values, increases the feeling of energy and heart rate [15, 46]. Moreover this can be explained by factors such as the tempo of the music or the type of music. In another study that supports our findings, Eliakim et al. [9] showed in their study that during the WAnT, the highest power (Ppeak) was achieved after warm up with music. Similarly Brooks et al. [28] aimed to examine whether music had a substantial impact on performance enhancement. A significant difference in anaerobic performance has been found when motivational music is played. Peak, average, and overall anaerobic power characteristics were all shown to be substantially different from when no music was played, with a reduction in power over time (*p* < 0.01). On every metric incorporating motivational music, performance values were much higher, as indicated [28]. Nakamura et al. [15] evaluated the effects of two types of music (preferred and non-preferred) on exercise distance, heart rate (HR), and perceived exertion rating during a high-intensity cycling exercise (RPE). According to the results of the study, the RPE for non-preferred music was greater than for other circumstances.

Jarraya et al. [4] investigated the impact of using music to warm up on short-term peak performance. Perceived effort rate (RPE) was measured during a 30-s WAnT following a 10-min warm-up with or without music in a study involving nine young male athletes (MWU). Although RPE and fatigue index were not modified by MWU, the power output during the Wingate test was considerably greater after MWU than after NMWU. These studies indicate that music has a positive impact on Wingate performance measures. Several published research indicate that music has little effect on Wingate performance measures. Isik et al. [44] investigated the influence of music on motivation during the Wingate Anaerobic Test (WAnT). There is a statistically significant difference in favor of conditioning with music in terms of maximal anaerobic power, maximum anaerobic capacity, relative anaerobic power, relative anaerobic capacity, and fatigue index, as determined by WAnT tests with and without music. There was no statistically significant difference in the minimum values of anaerobic power.

Ballmann et al. [14] investigated the impact of listening to 2 different (preferred and unpreferred) types of music on repeated sprint performance. Participants in a balanced crossover study design made two different visits. At each visit, participants listened to either preferred or non-preferred music and performed 3 × 15 s Wingate Anaerobic Tests separated by 2-min active recovery periods. Average power, anaerobic capacity, and total effort were not statistically significantly different between preferred and non-preferred music conditions after at least 48 h of recovery. The participants' average heart rate did not change. These results differ from our findings. The main reason for the differences may be the type of music or visual stimuli in the research protocol.

Chtourou et al. [45] examined the impact of listening to music while warm up for the WAnT on variations in daily power output. Physical education teachers participated in four WAnT following a 10-min warm-up with and without music at different times of the day (07:00 and 17:00) after a 10-min warm-up with and without music. According to the results of the study, peak and average power increased after listening to music from morning until afternoon. The mechanism by which music positively affects performance is unknown.

When the studies examining the impacts of visual feedback on performance are reviewed, it is reported that visual feedback has beneficial effects on performance [47, 48]. Stastny et al. [20] investigated the influence of visual feedback on the power output of hockey players using an intermittent WAnT (AnWT6 × 6) with a 1:1 work-rest ratio that was particular to hockey players. It has been observed that visual feedback can favourably influence power output in the unfatigued condition (stage 1), but plays no meaningful function in the WAnT expression of excessive neuromuscular fatigue (stage 6). These studies are similar to our findings. Arnăutu and Hanțiu [29] proposed to analyze the effect of using equipment that provides athletes with visual feedback on the yield in boxing training. The results of this study show us that the motivation elements and the visual feedback introduced in the case of the experiment group had the effect of increasing the number of punches transmitted in the boxing bag, compared to the control group. Hopper et al. [31] investigated the impact of visual feedback on power performance during the leg press in sixteen elite female field hockey players between the ages of 16 and 27. Visual feedback has a considerable favorable effect on the power performance of elite female field hockey players, according to the study's findings. Kim and Kramer [49] investigated the knee extensor torques of groups with and without visual feedback over the course of two weeks in their study. They concluded that the knee extensor torques produced during visual feedback were greater than those produced by groups without visual feedback. Kellis and Baltzopoulos [50] investigated the effect of maximum moment measurements of knee extensors and flexors during isokinetic eccentric activations with and without visual feedback. Consequently, they found that the mean elongation peak moments at 30°/sec and 150°/sec in evaluations with visual feedback were nearly 7.2% and 6.4% greater, respectively, than in data without visual feedback. The knee flexor moment increased by 8.7 and 9 percent at slow and high speeds, respectively.

There are several limitations highlighted in the study. Initially, a small number of single-sex volunteers were recruited for this study. One of our limitations was that the participants could listen to preferred music and non-preferred music. Another limitation was that different types of music could be used. In addition, the variable of Rating of perceived exertion and heart rate variable could be tested. Athletes from a variety of sports, such as judokas, kickboxers, volleyball players, and basketball players, could have volunteered for this study. Therefore, the results cannot be generalized to all sports branches.

## Conclusion

Coaches, sports scientists, and researchers can provide visual feedback with music (120–140 bpm) as a warm-up program prior to the WAnT, as it can have a positive impact on performance and help athletes achieve peak power. The positive development of Wingate's performances was facilitated by a warm-up consisting of visual feedback and music performed by futsal players. Positive responses to MV indicated that futsal players' WAnT values, including PPO, RPPO, MPO, RMPO, and FI, improved more than with other protocols. Coaches should perform tests in multiple situations to establish consistent responses to various visual feedback or music warm-ups and thus create their own individualized, optimal warm-ups. However, the results clearly indicate a positive effect, so additional research is required to determine the optimal warm-up procedures for futsal players.

## Data Availability

The data presented in this study are available on website: https://osf.io/ckte5/ with Identifier: https://doi.org/10.17605/OSF.IO/CKTE5.

## References

[1] Belkhir Y, Rekik G, Chtourou H, Souissi N (2019). Listening to neutral or self-selected motivational music during warm-up to improve short-term maximal performance in soccer players: effect of time of day. Physiol Behav.

[2] Chtourou H, Jarraya M, Aloui A, Hammouda O, Souissi N (2012). The effects of music during warm-up on anaerobic performances of young sprinters. Sci Sports.

[3] Fox RP, Michael TJ, Weideman CA, Hanson NJ (2019). Effect of listening to music during a warmup on anaerobic test performance. Sport Sci Health.

[4] Jarraya M, Chtourou H, Aloui A, Hammouda O, Chamari K, Chaouachi A (2012). The effects of music on high-intensity short-term exercise in well trained athletes. Asian J Sports Med.

[5] Eken Ö, Clemente FM, Nobari H (2022). Judo specific fitness test performance variation from morning to evening: specific warm-ups impacts performance and its diurnal amplitude in female judokas. BMC Sports Sci Med Rehabil.

[6] Kafkas A, Eken Ö, Kurt C, Kafkas ME (2019). The effects of different stretching and warm-up exercise protocols on 50-meter swimming performance in sub-elite women swimmers. Isokinet Exerc Sci.

[7] Patti A, Giustino V, Cataldi S, Stoppa V, Ferrando F, Marvulli R (2022). Effects of 5-week of FIFA 11+ warm-up program on explosive strength, speed, and perception of physical exertion in elite female futsal athletes. Sports.

[8] Abade E, Sampaio J, Gonçalves B, Baptista J, Alves A, Viana J (2017). Effects of different re-warm up activities in football players’ performance. PLoS ONE.

[9] Eliakim M, Meckel Y, Nemet D, Eliakim A (2007). The effect of music during warm-up on consecutive anaerobic performance in elite adolescent volleyball players. Int J Sports Med.

[10] Eken Ö, Yagin FH, Eken I, Gabrys T, Knappova V, Bayrakdaroglu S (2022). Diurnal variation in Uchikomi fitness test performance: Influence of warm-up protocols. Front Psychol.

[11] McGowan CJ, Pyne DB, Thompson KG, Rattray B (2015). Warm-up strategies for sport and exercise: mechanisms and applications. Sport Med.

[12] Febbraio MA, Carey MF, Snow RJ, Stathis CG, Hargreaves M (1996). Influence of elevated muscle temperature on metabolism during intense, dynamic exercise. Am J Physiol Integr Comp Physiol.

[13] Castañeda-Babarro A, Marqués-Jiménez D, Calleja-González J, Viribay A, León-Guereño P, Mielgo-Ayuso J (2020). Effect of listening to music on Wingate anaerobic test performance. A systematic review and meta-analysis. Int J Environ Res Public Health.

[14] Ballmann CG, McCullum MJ, Rogers RR, Marshall MM, Williams TD (2018). Effects of preferred vs. nonpreferred music on resistance exercise performance. J Strength Cond Res.

[15] Nakamura PM, Pereira G, Papini CB, Nakamura FY, Kokubun E (2010). Effects of preferred and nonpreferred music on continuous cycling exercise performance. Percept Mot Skills.

[16] Hayakawa Y, Takada K, Miki H, Tanaka K (2000). Effects of music on mood during bench stepping exercise. Percept Mot Skills.

[17] Atan T (2013). Effect of music on anaerobic exercise performance. Biol Sport.

[18] Simpson SD, Karageorghis CI (2006). The effects of synchronous music on 400-m sprint performance. J Sports Sci.

[19] Pujol TJ, Langenfeld ME (1999). Influence of music on Wingate anaerobic test performance. Percept Mot Skills.

[20] Stastny P, Tufano J, Kregl J, Petr M, Blazek D, Steffl M (2018). The role of visual feedback on power output during intermittent Wingate testing in ice hockey players. Sports.

[21] Minahan C, Chia M, Inbar O (2007). Does power indicate capacity? 30-s Wingate anaerobic test vs. maximal accumulated O_2_ deficit. Int J Sports Med.

[22] Bertuzzi R, Kiss MAPDM, Damasceno M, Oliveira RSF, Lima-Silva AE (2015). Association between anaerobic components of the maximal accumulated oxygen deficit and 30-second Wingate test. Braz J Med Biol Res.

[23] Madrid B, Pardono E, Farias DL de, Asano RY, Silva RJ dos S, Simões HG. Reprodutibilidade do teste anaeróbio de Wingate em ciclistas. Motricidade. 2013; 10.6063/motricidade.9(4).1130.

[24] Driss T, Vandewalle H (2013). The measurement of maximal (anaerobic) power output on a cycle ergometer: a critical review. Biomed Res Int.

[25] Gülü M, Akalan C (2021). A new peak-power estimation equations in 12 to 14 years-old soccer players. Medicine (Baltimore).

[26] Güçlüöver A, Gülü M (2020). Developing a new muscle power prediction equation through vertical jump power output in adolescent women. Medicine (Baltimore).

[27] Zupan MF, Arata AW, Dawson LH, Wile AL, Payn TL, Hannon ME (2009). Wingate anaerobic test peak power and anaerobic capacity classifications for men and women intercollegiate athletes. J Strength Cond Res.

[28] Brooks K, Brooks K, Brooks WD (2009). Difference in Wingate power output in response to music as motivation. Med Sci Sport Exerc.

[29] Arnăutu G-C, Hanțiu I (2021). The effect of introducing visual feedback on sports training. Stud Univ Babeş-Bolyai Educ Artis Gymnast.

[30] Baltzopoulos V, Williams JG, Brodie DA (1991). Sources of error in isokinetic dynamometry: effects of visual feedback on maximum torque measurements. J Orthop Sport Phys Ther.

[31] Hopper DM, Axel Berg MA, Andersen H, Madan R (2003). The influence of visual feedback on power during leg press on elite women field hockey players. Phys Ther Sport.

[32] Campenella B, Mattacola CG, Kimura IF (2000). Effect of visual feedback and verbal encouragement on concentric quadriceps and hamstrings peak torque of males and females. Isokinet Exerc Sci.

[33] Faul F, Erdfelder E, Lang A-G, Buchner A (2007). G*Power 3: a flexible statistical power analysis program for the social, behavioral, and biomedical sciences. Behav Res Methods.

[34] Fox EL, Bowers RWFM (1988). The physiological basis of physical education and athletics.

[35] Souissi N, Gauthier A, Sesboüé B, Larue J, Davenne D (2004). Circadian rhythms in two types of anaerobic cycle leg exercise: force-velocity and 30-s Wingate tests. Int J Sports Med.

[36] Karvonen MJ, Kentala EMO (1957). The effects of training on heart rate: a longitudinal study. Ann Med Exp Biol Fenn.

[37] Nes BM, Janszky I, Wisløff U, Støylen A, Karlsen T (2013). Age-predicted maximal heart rate in healthy subjects: the HUNT fitness study. Scand J Med Sci Sports.

[38] Edworthy J, Waring H (2006). The effects of music tempo and loudness level on treadmill exercise. Ergonomics.

[39] Karageorghis C, Jones L, Stuart D (2008). Psychological effects of music tempi during exercise. Int J Sports Med.

[40] Hoffman JR, Kang J. Evaluation of a new anaerobic power testing system. J strength Cond Res. 2002;16:142–8. http://www.ncbi.nlm.nih.gov/pubmed/11834120. 11834120

[41] Bradley AL, Ball TE (1992). The Wingate test: effect of load on the power outputs of female athletes and nonathletes. J Strength Cond Res.

[42] Bar-Or O (1987). The Wingate anaerobic test. Sport Med.

[43] Chen C-C, Chen Y, Tang L-C, Chieng W-H (2022). Effects of interactive music tempo with heart rate feedback on physio-psychological responses of basketball players. Int J Environ Res Public Health.

[44] Işık Ö, Ersöz Y, Pazan M, Ocak Y (2015). The effect of motivational music on Wingate anaerobic test performance. Int J Hum Sci.

[45] Chtourou H, Chaouachi A, Hammouda O, Chamari K, Souissi N (2012). Listening to music affects diurnal variation in muscle power output. Int J Sports Med.

[46] Lingham J, Theorell T (2009). Self-selected, “favourite” stimulative and sedative music listening—how does familiar and preferred music listening affect the body?. Nord J Music Ther.

[47] Carlson AJ, Bennett G, Metcalf J (1992). The effect of visual feedback in isokinetic testing. Isokinet Exerc Sci.

[48] Hobbel SL, Rose DJ (1993). The relative effectiveness of three forms of visual knowledge of results on peak torque output. J Orthop Sport Phys Ther.

[49] Kim HJ, Kramer JF (1997). Effectiveness of visual feedback during isokinetic exercise. J Orthop Sport Phys Ther.

[50] Kellis E, Baltzopoulos V (1996). Resistive eccentric exercise: effects of visual feedback on maximum moment of knee extensors and flexors. J Orthop Sport Phys Ther.

